# An open-source toolbox for measuring vocal tract shape from real-time magnetic resonance images

**DOI:** 10.3758/s13428-023-02171-9

**Published:** 2023-07-28

**Authors:** Michel Belyk, Christopher Carignan, Carolyn McGettigan

**Affiliations:** 1 Department of Psychology, Edge Hill University, Ormskirk, UK; 2 Department of Speech Hearing and Phonetic Sciences, University College London, London, UK

**Keywords:** Real-time MRI, Vocal tract imaging, Magnetic resonance imaging, Software

## Abstract

Real-time magnetic resonance imaging (rtMRI) is a technique that provides high-contrast videographic data of human anatomy in motion. Applied to the vocal tract, it is a powerful method for capturing the dynamics of speech and other vocal behaviours by imaging structures internal to the mouth and throat. These images provide a means of studying the physiological basis for speech, singing, expressions of emotion, and swallowing that are otherwise not accessible for external observation. However, taking quantitative measurements from these images is notoriously difficult. We introduce a signal processing pipeline that produces outlines of the vocal tract from the lips to the larynx as a quantification of the dynamic morphology of the vocal tract. Our approach performs simple tissue classification, but constrained to a researcher-specified region of interest. This combination facilitates feature extraction while retaining the domain-specific expertise of a human analyst. We demonstrate that this pipeline generalises well across datasets covering behaviours such as speech, vocal size exaggeration, laughter, and whistling, as well as producing reliable outcomes across analysts, particularly among users with domain-specific expertise. With this article, we make this pipeline available for immediate use by the research community, and further suggest that it may contribute to the continued development of fully automated methods based on deep learning algorithms.

## Introduction

**Fig. 1:**
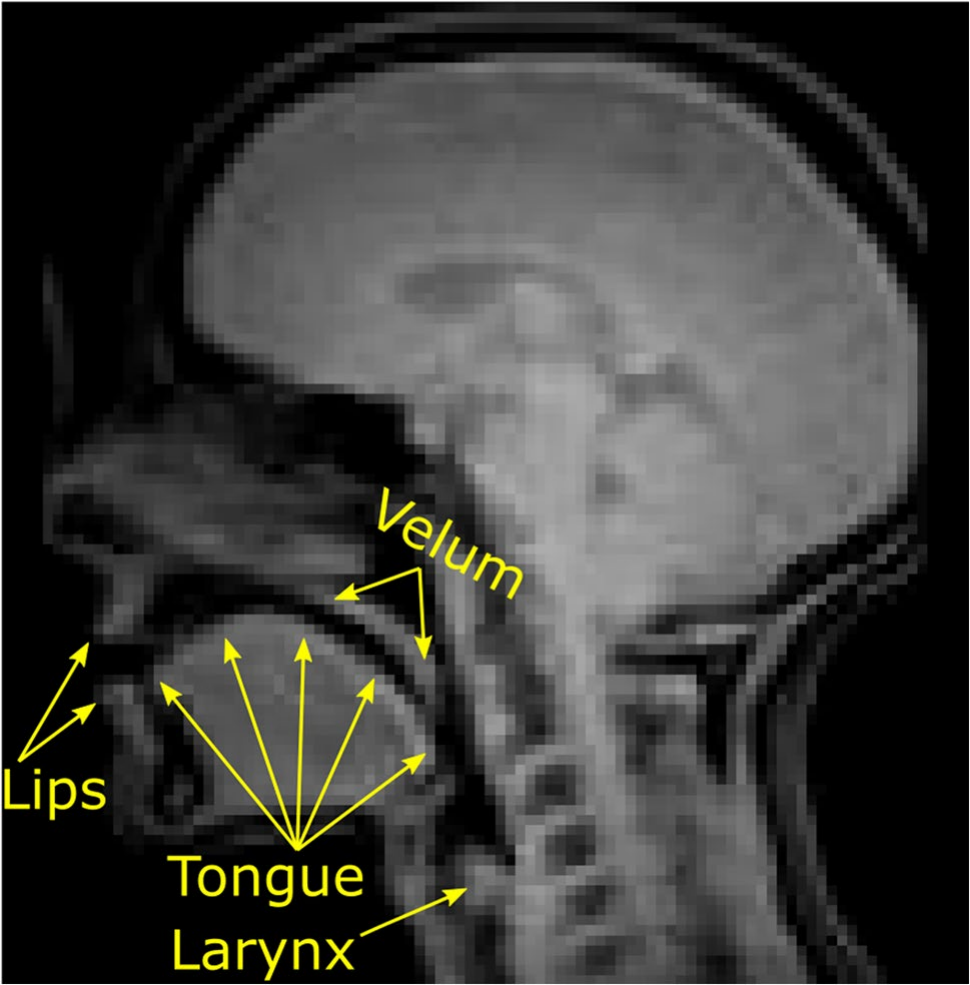
Sample frame from one rtMRI run. The greyscale image covers a mid-sagittal slice from a single time point with soft tissue shown as light and air or bone shown as dark pixels. Labile structures of the vocal tract are labelled for convenience, and the vocal tract constitutes the negative space between them

Real-time magnetic resonance imaging (rtMRI) is a technique for producing dynamic videos of the internal structures of the body (Zhang et al., 2010). It is frequently applied by speech scientists to study movements of the internal structures of the head that shape speech sounds, which are not easily amenable to external observation. The technique has been used to study a broad range of behaviours including the articulatory movements of speech (Belyk et al., 2019; Carey et al., 2017; Carignan et al., 2020; Miller et al., 2014; Narayanan et al., 2014; Wiltshire et al., 2021), vocal registers of singers (Echternach et al., 2010; Lynn et al., 2021), and vocal expressions of emotion (Belyk & McGettigan, 2022), as well as non-speech movements such as swallowing (Mills et al., 2020; Olthoff et al., 2014; Zhang et al., 2012) and beat-boxing (Proctor et al., 2013). In typical uses of the technique, a single mid-sagittal slice through the head and neck forms an image that transects the vocal tract (see Fig. 1). Repeated measurements taken on the order of milliseconds compose the frames of a videographic record.

Speech scientists use rtMRI to study movements of the lips, tongue, velum, and larynx. Together, these structures change the shape of the vocal tract, which determines which of the broad range of sounds from the human repertoire a speaker will produce at any particular moment (Fant, 1960; Titze, 2008). These structures are highly labile, having an extensive range of possible motions and configurations, which are executed in rapid and coordinated succession. The study of this system is made tractable by the fact that it is deterministic: there are systematic and quantifiable relationships between the shape of the vocal tract, the physical acoustics of the sounds that it produces, and the perceptions of the people who hear it (Fant, 1960; Titze, 2008). The principal challenge has been that these anatomical structures and their movements are difficult to observe. However, non-invasive imaging through technologies such as rtMRI (Zhang et al., 2010) provide a means of quantifying vocal phenomena that were not otherwise easily quantifiable.

A principal difficulty for the use of this technology is in deriving anatomically meaningful measurements from the complex images produced by rtMRI. The standard use case has been to choose a small number of theoretically interesting features and measure the sizes and distances between anatomical structures on a small subset of the images that are collected (Echternach et al., 2010, 2011, 2014; Lammert et al., 2011). While this approach has yielded several significant insights, it also discards vast amounts of potentially informative data that are present in the images. It is also extremely laborious, which prohibits the use of the larger sample sizes that have become a standard of good scientific practice for other disciplines.

A promising avenue for development has come from the application of machine learning, and deep learning algorithms in particular (Goodfellow et al., 2016). This is a field of statistics that continues to develop rapidly, and several implementations have been proposed for applications to rtMRI (e.g., Asadiabadi & Erzin, 2020; Bresch & Narayanan, 2009; Eslami et al., 2020; Labrunie et al., 2018; van Leeuwen et al., 2019; Mannem & Ghosh, 2021; Pandey & Sabbir Arif, 2021; Ruthven et al., 2021; Silva & Teixeira, 2015; Somandepalli et al., 2017; Takemoto et al., 2019; Valliappan et al., 2019). Deep-learning-based approaches can yield machine-generated traces of the vocal tract which are sufficiently accurate to be useful for scientific measurements. However, to our knowledge, none have been demonstrated to generalise well to new datasets (i.e., those produced by behaviours, imaging hardware, and pulse sequences not observed in the training data). Hence, while these advances are of intense theoretical interest, they have not yet presented a path forward for practising behavioural scientists.

One of the key obstacles towards generalisability is the limited availability of validated vocal tract traces from which to train the algorithms. Indeed, existing implementations have sampled from a narrow range of available corpora composed of small numbers of speakers and tasks that were recorded from a single imaging centre (e.g., Narayanan et al. 2014). While these attributes make a corpus fertile testing ground for the development of new techniques, they do little to promote the application of these techniques to novel datasets. Hence, despite impressive advances in the engineering of deep learning architectures as applied to rtMRI, they have yet to be widely adopted in practice.

To date there is therefore no deep learning solution that practising scientists can confidently apply to new experimental data. While development continues on deep learning methods, several research groups have developed idiosyncratic pipelines that facilitate measurement (Belyk et al., 2019; Carignan et al., 2020; Kim et al., 2014). These methods use signal processing approaches such as tissue classification or edge detection to partially automate measurement, supplemented by user input that provides the domain-relevant expertise of the analyst. Ideally such methods should strike a balance between precision and labour efficiency*—*they should also have a demonstrable capacity to generalise effectively to new use cases. However, to date no such demonstration of generalisability has been forthcoming.

Here, we describe a practical signal processing pipeline for taking useful scientific measurements from mid-sagittal rtMRI images. We provide an overview of the pipeline and report validation tests which demonstrate that (1) the method can be generalised to new use case, and (2) can be successfully applied by users outside of the development team. We make this pipeline available for immediate use by the research community, and discuss how it may contribute to the further development of fully automated methods based on deep learning algorithms.

## Implementation

**Fig. 2:**
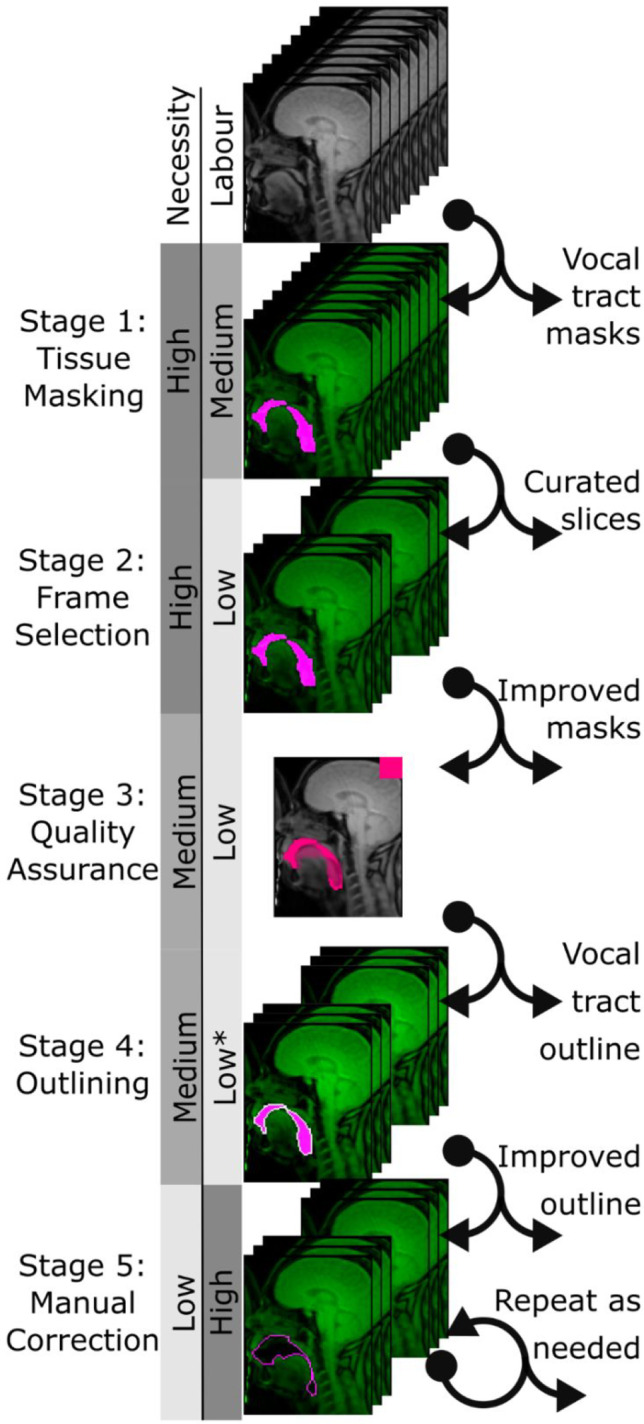
Overview of vocal tract morphology pipeline from raw image data through processing stages 1–5. Each stage in the sequence produces inputs needed for later stages as well as outputs which researchers may find useful for further analysis. Depending on the research question being addressed, researchers may choose to forgo later processing stages. The key on the left-hand side provides a rough index of how necessary each stage is for producing useful measurements, and the degree of labour that each step requires. Researchers with very large datasets may consider forgoing the more labour-intensive late stages; however, the best outcomes will be achieved by completing all processing stages. Note: The example shown in stage 5 depicts a different image frame for illustrative purposes. *Stage 4 requires little human labour but may be computationally intensive for some datasets

This pipeline is implemented as a series of MATLAB scripts (with dependencies from the Image Processing Toolbox and the Statistics and Machine Learning Toolbox) which execute the stages of this processing pipeline (see Fig. 2) in series. Each script generates outputs that are useful either for diagnosing analysis quality or as the inputs to subsequent stages of analysis. Code and documentation can be retrieved from the Open Science Framework (https://osf.io/hm6zp/).

## Overview

Our approach uses simple tissue classification constrained to pixels that the analyst has identified are likely to contain the vocal tract and surrounding tissue. Gross input provided by the analyst on the basis of one frame is used as a seed for the analysis of an entire imaging run. This procedure is designed to reduce labour-intensiveness, while retaining input from domain-specific expertise.

The task that most researchers will wish to accomplish effectively reduces to edge detection—that is, finding the boundaries between tissue and air that outline the vocal tract. A mid-sagittal slice of the head and neck contains many edges, only a minority of which are of interest. However, while the vocal tract changes shape, it does not generally change position within the MRI scanner, nor do the features of interest to researchers.

The pipeline that we present takes the following steps:

1. *Spatially informed tissue masking*
  - *User manually identifies a region of interest on the basis of one frame.*
  - *Tissue classification proceeds for all frames within the same imaging run.*
2. *Frame selection*
  - *Time points of interest are identified from user-provided logfiles.*
  - *Remaining time points are discarded for computational and labour efficiency.*
3. *Quality assurance*
  - *Summary statistics identify spatial locations with high rates of suspected classification errors across an imaging run.*
  - *User updates region of interest as needed.*
4. *Vocal tract outlining*
  - *Tissue masks are converted to outlines.*
  - *Connecting lines drawn automatically between vocal tract cavities if required.*
5. *Manual correction*
  - *User review on a frame-by-frame basis with opportunities to draw or erase individual features.*
  - *Perform and repeat as appropriate to balance precision and labour expenditure.*

While each stage must be followed in order (see overview in Fig. 2), analysts may choose to forgo later stages depending on their research needs. For example, researchers with very large datasets may wish to omit the final manual correction stage or apply it to only a randomly sampled subset of the data in order to assess measurement error rather than correct it exhaustively. These descriptions are intended as a conceptual demonstration, and readers are referred to the user manual for guidance on executing each step.

### Stage 1: Spatially informed tissue masking

The goal of this processing pipeline is to locate image pixels that outline the vocal tract. However, the majority of edges within a mid-sagittal MR image correspond to non-vocal tract structures and tissues, such as the scalp, brain, and spinal cord. The pipeline therefore requests user input to identify a region of interest that is likely to contain the vocal tract and surrounding tissue. This area of interest is specified on the basis of a single image and then used to restrict tissue classification across all images within a run.

An initial estimate of the region of the space that is likely to contain the vocal tract is identified by searching for pixels whose intensity values are highly variable across the rtMRI run. Pixels with fixed values are likely to contain static structures of little interest (e.g., the skull or air outside the vocal tract), while high variance pixels are likely to alternate between air and soft tissue; that is, they alternate between containing the labile structures that outline the vocal tract and the air within the vocal tract itself. Assuming that the images are relatively free of time varying noise, such a variance map roughly follows the course of the vocal tract provides a convenient starting point from which to identify a vocal tract region of interest. However, this automatic method is likely either to overreach into adjacent structures of no interest to speech research (e.g., into the vertebrae) or to exclude pixels that contain fixed structures that nonetheless form part of the outline of the vocal tract (e.g., the palate). A graphical user interface is therefore provided to manually refine the area of interest. This procedure is repeated once for each imaging run to produce a mask of candidate pixels that may contain the vocal tract at any time point during the run.

**Fig. 3:**
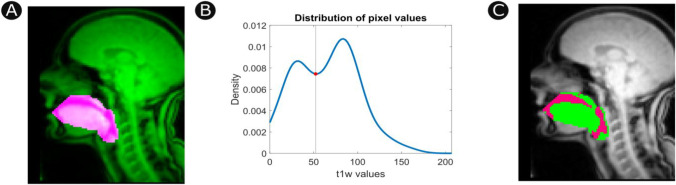
Spatially constrained tissue classification. **A** The search area for the vocal tract and surrounding tissue (pink) is identified with user input. **B** The distribution of T1-weighted pixel values in the search area is bimodal. The local density minimum is identified as a threshold for simple tissue classification (vertical line). **C** Pixels with T1 intensity values above the classification threshold are classified as tissue (green), while pixels below the threshold are classified as air (magenta). Classification is applied iteratively across all frames within an rtMRI run

Finally, simple tissue classification is performed within the area of interest (see Fig. 3). The distribution of pixel values within the analyst-specified mask is expected to be bimodal, reflecting the underlying source of T1-weighted signals from air and soft tissue, respectively. The local density minimum is identified as a threshold for simple tissue classification. Applying this threshold to each frame in the rtMRI run results in a binary mask marking pixels that contain the vocal tract.

### Stage 2: Frame selection

Depending on the research question, analysts may not be equally interested in all time points within a run. For example, time points that capture swallowing or the inspiratory phase of speech may not be relevant to experiments on speech articulation. This step reads logfiles that indicate which frames should be retained for further analysis and discards the remainder. A Praat (Boersma & Weenink, 2019) script is provided along with the processing pipeline, which produces logfiles from MRI-aligned audio recordings if they are available. For each segment of interest, these logfiles note the onset frame and offset frame, as well as an optional text label. The Praat helper script also generates several useful acoustical measurements such as fundamental frequency, duration, and sound intensity level, though interpretation of these acoustical measures may merit caution depending on the quality of in-scanner audio recording.

### Stage 3: Quality assurance

**Fig. 4:**
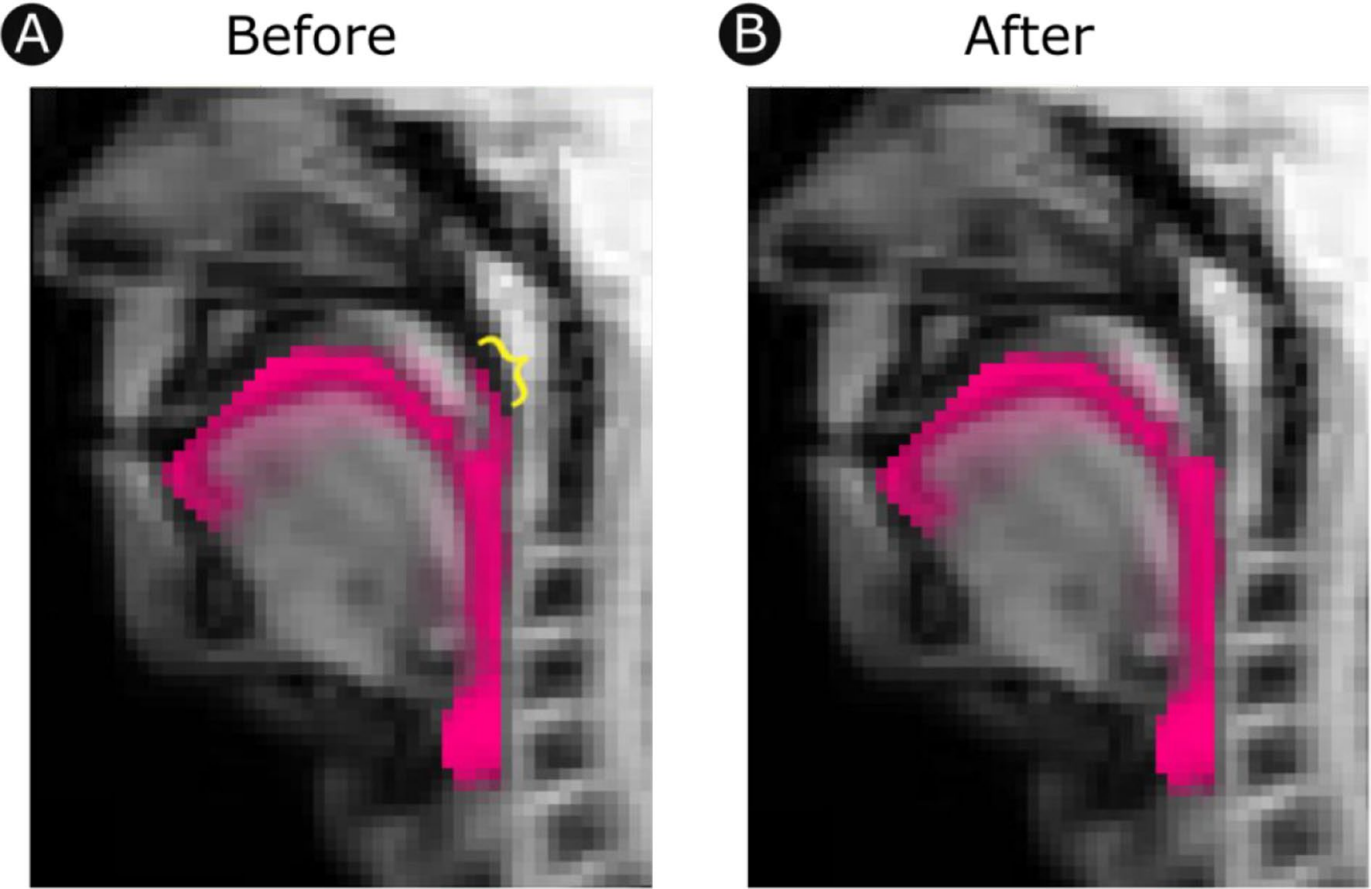
Quality assurance. Users inspect a map depicting the proportion of frames in which each pixel was classified as vocal tract (pink). Domain- and project-specific knowledge is used to further exclude pixels that are routinely misclassified relative to the needs of the project. **A** Vocal tract map including a cluster of pixels above the velum that were not deemed relevant by the analyst (indicated by the yellow curly brace). **B** The same map with the error-prone pixels excluded. This exclusion is specified once per run and extrapolated to all frames within it

User input from the initial tissue classification stage may produce errors if the area of interest extends outside of the contiguous oropharyngeal vocal tract cavity. In this stage, a map of the proportion of images in which each pixel was classified as vocal tract is overlaid onto a reference image. Pixels are tinted pink on a scale ranging from translucent (pixel is never classified as vocal tract) to opaque (pixel is always classified as vocal tract); the resulting map provides an easy means of identifying pockets of space that are occasionally misclassified. Such pixels are highlighted as islands of pink among an ocean of anatomical grey (see Fig. 4). Analysts are shown one quality assurance map for each imaging run, and can use a graphical user interface to manually exclude pixels from further analysis.

### Stage 4: Vocal tract outlining

Depending on the researcher’s data analysis plans, the binary masks produced by the steps described above may be sufficient. However, in many cases the researcher may require an outline of the vocal tract that captures the outer boundaries of its shape rather than a mask of its internal area.

**Fig. 5:**
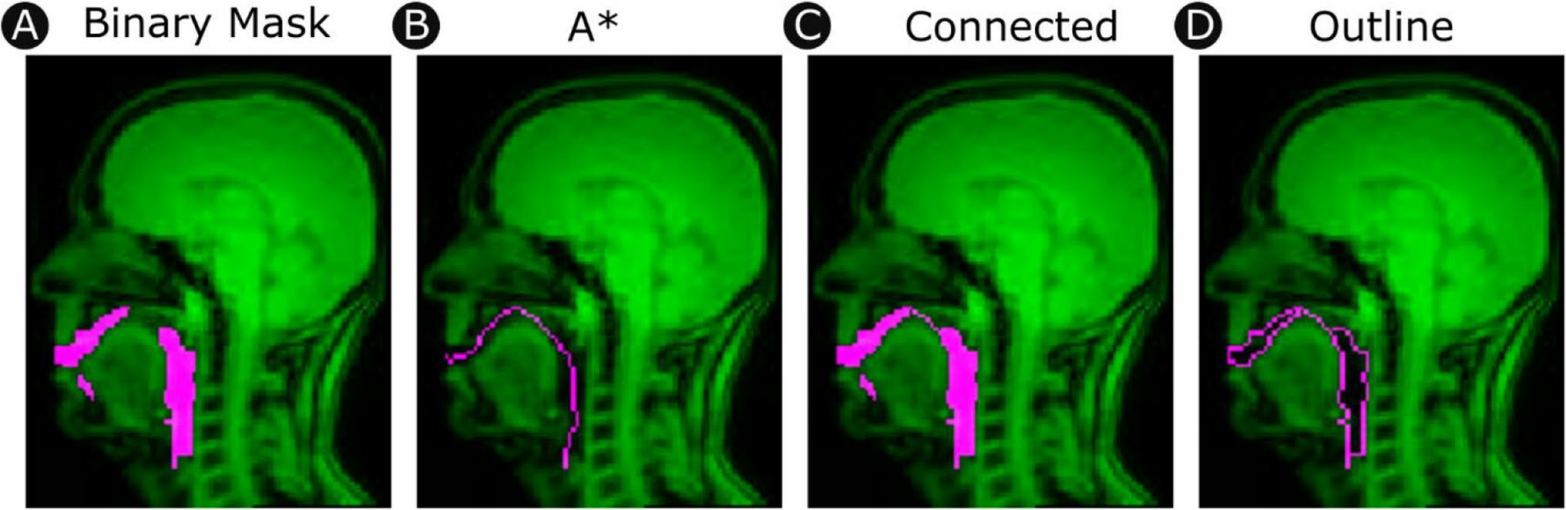
Vocal tract outlines. **A** The binary mask identifying pixels as belonging to the vocal tract. **B** The mask is navigated by the A* method, which finds a parsimonious path from larynx to lips. **C** This path is drawn as a connector between otherwise isolated vocal tract cavities. **D** A single continuous outline of the vocal tract can then be drawn to capture the morphology of the vocal tract

In many instances the vocal tract may be bisected into multiple discontinuous cavities by the action of the articulators (e.g., stop consonants which are articulated by complete obstruction of the vocal tract). Indeed, these articulations are frequently of primary interest to the researcher, and a meaningful analysis will require a connecting line to be drawn between vocal tract cavities following a biologically plausible path (see Fig. 5).

“A*” is an algorithm for machine navigation that finds the shortest available path through a maze of valid locations and possibly weighted to prefer some paths over others (Hart et al., 1968). This step configures A* to walk from the larynx to the lips, weighting each pixel with the inverse of the T1-weighted signal at its location. Hence, the algorithm seeks a parsimonious path through the vocal tract by following the dim, if not altogether dark, pixels at sites of constriction. This path acts as a connection between vocal tract cavities so that the vocal tract may be modelled as a whole rather than as discrete cavities. In most cases these connectors will be biologically sound, but this is not guaranteed, and care should be taken to verify that outputs are as expected.

### Stage 5: Manual correction

**Fig. 6:**
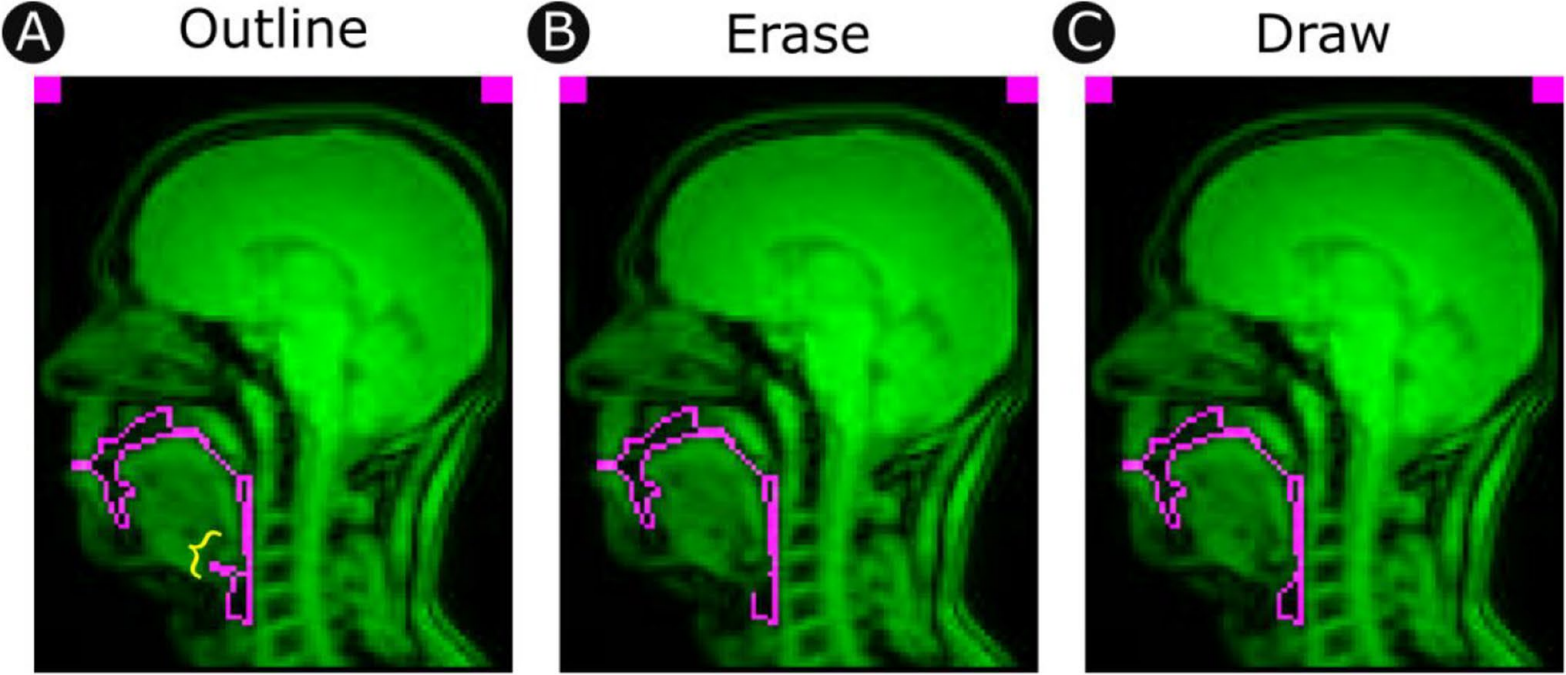
Manual correction. **A** In this example an undesirable feature is identified near the tongue root (highlighted by yellow curly brace). **B** A simple graphical user interface allows the analyst to erase and **C** redraw segments of the outline

Several stages in this pipeline have provided opportunities for input from an informed analyst. These procedures are an important part of the pipeline which help to ensure the quality of the measurements and their appropriateness to individual research questions. At this final stage, the analyst can review vocal tract outlines frame-by-frame, draw, erase, and retrace as needed (see Fig. 6).

## Validation

### Alpha test: Generalisability to new datasets

#### Procedure

**Fig. 7:**
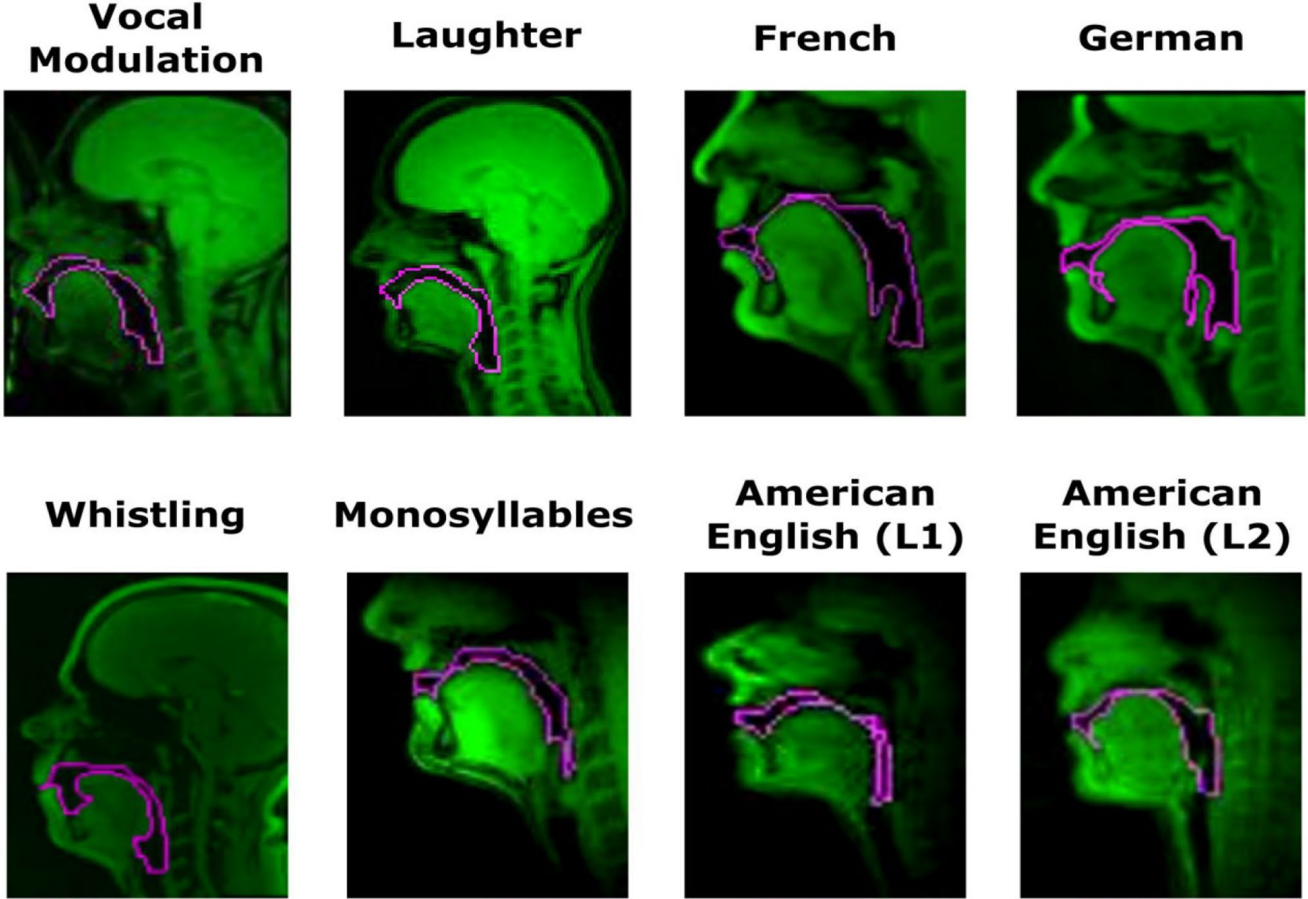
Representative sample traces (pink) from each of the four datasets on which the pipeline was tested. Variation in T1-signal intensity (green) reflects image quality differences between the underlying datasets. Image quality varies considerably between datasets, which may influence signal processing outcomes. In some instances, the imaging data were enhanced by project-specific data pre-processing, which is standard procedure in other disciplines that make use of MRI. These traces are prior to manual correction, such that further improvements could be achieved as and when needed

We tested the generalisability of the pipeline by having a single analyst (MB) apply the pipeline to eight sets of sample data covering a range of behaviours that may interest speech scientists. These were selected on the basis of availability and to sample across a breadth of available hardware, software, and wetware—the biological component that is being imaged. Hence, one imaging run from one participant was analysed from each dataset. These samples were composed of 500–3216 sagittal slices per run, of which 113–2808 were analysed after discarding time points that were unlikely to be of interest (i.e., retaining time points in which audible sound was produced by the participant as identified by synchronised audio data). These samples covered a range of speech and non-speech behaviours across a range of research disciplines (see Table 1), including spoken monosyllables in British English, connected speech in German (Carignan et al., 2020), French (Isaieva et al., 2021), American English as spoken by a native (L1) speaker (Narayanan et al., 2014), and American English spoken by a non-native speaker (Lim et al., 2021), as well as non-speech vocal behaviours including vocal size exaggeration (Belyk et al., 2022), laughter (Belyk & McGettigan, 2022), and whistling (Belyk et al., 2019). This sample reflects the natural variation in imaging parameters, and correspondingly in image quality, that analysts may face in practical application (see Fig. 7).

**Table 1:** Data sources and basic image dimensions for alpha test datasets

#	Site	Behaviour	MRI hardware	FPS	mm	Matrix Size
1	A	Vocal size exaggeration	Siemens 3T TIM Trio	8	2.5 x 2.5 x 10	112 x 90
2	A	Laughter	Siemens 3T TIM Trio	8	2.5 x 2.5 x 10	112 x 90
3	B	Whistling	Siemens 3T Magnetom Prisma Fit	16.67	2 x 2x 8	128 x 128
4	C	German	Siemens 3T Magnetom Prisma Fit	50	1.41 x 1.41 x 8	136 x 136
5	D	French	Siemens 3T Magnetom Prisma	50	1.6 x 1.6 x 8	136 x 136
6	E	American English (L1)	GE 1.5T Signa Excite HD	23.18	2.9 x 2.9 x 5	68 x 68
7	E	American English (L2)	GE 1.5T Signa Excite HD	83.28	2.4 x 2.4 x 6	84 x 84
8	F	British Monosyllables	Siemens 3T Magnetom Prisma	33	2 x 2 x 8	128 x 128

We note that the pulse sequences underlying image acquisition also vary between scanning sites and refer readers to the original sources for further details. The behaviours sampled represent a range from monosyllabic words to connected speech in a range of languages (German, French, British English, American English) at varied language proficiencies (L1, L2) as well as non-speech vocal behaviour (whistling, laughter, size exaggeration). Datasets #1–7 are re-analyses of published data. Dataset #8 is pilot data collected by the authors. FPS: frames per second as a metric of temporal resolution; mm: spatial resolution in millimetres within the sagittal plane (*y*, *z*) and slice thickness (*x*), respectively.

#### Outcome

We demonstrate that data covering a range of scanning sites, behaviours, and imaging parameters were well tolerated by the analysis pipeline. Sample traces from each dataset are presented in Fig. 7. The most substantive obstacle was variation in the degree to which data were affected by intensity inhomogeneity. T1-weighted MRI images are prone to contamination by intensity gradients such that the same category of tissue may appear brighter in one part of the image than in others (Sled & Pike, 1998). In processing the raw images, intensity inhomogeneity strongly interfered with tissue segmentation in Stage 1 of this pipeline. However, it was found that pre-processing the raw images to minimise intensity inhomogeneity mitigated these issues. Such pre-processing steps are a standard item of consideration in other disciplines that make use of T1-weighted MRI, for example cortical thickness measurements in brain imaging (Haast et al., 2018). Supplementary methods for implementing inhomogeneity correction are also included in the distribution package. We note that whether any, and if so which, pre-processing steps may be beneficial should be considered on a per-dataset basis.

### Beta test: Generalisability to new users

#### Procedure

In light of the signal processing pipeline’s reliance on user input, we tested how strongly outcomes varied between analysts. Seven analysts processed a single run from the vocal size exaggeration dataset (Belyk et al., 2022; Waters et al., 2021). Three analysts were vocal tract experts with extensive familiarity with mid-sagittal views of the vocal tract (authors MB, CC, CM), and of these only one had previous experience using the pipeline (MB). Four were speech and voice researchers familiar with the biomechanics of speech but with limited experience in vocal tract imaging. All analysts were provided with a work package including data, scripts, and documentation, but were not given further instructions.

Dice scores were computed as a measure of similarity between each pair of analysts (Dice, 1945). Dice scores are calculated as twice the number of features (i.e., pixels classified as vocal tract) that are shared by two sets of analyses divided by the sum of the number of features in each set (see Eq. 1). These calculations were carried out on the basis of outputs from Stages 1–3 (quality assurance), as Dice scores are more suitable to analyses of areas, which are outputted by these early stages, rather than the outlines which are produced by the later stages. In particular, Dice scores are based on the number of identical items, and hence would treat large disagreements in vocal tract outlines as equally problematic to small disagreements.

1 $$DiceScore=\frac{2\left|X\cap Y\right|}{\left|X\right|+\left|Y\right|}$$

A separate measure of inter-user disagreement was also examined at the level of individual sites along the outline of the vocal tract in order to provide a more nuanced view of the locations of potential disagreement based on the final output of the pipeline (i.e., after all opportunities for manual correction). The outlines of the vocal tract produced by each participant were modelled as continuous functions using the techniques of functional data analysis (Ramsay et al., 2009, 2017). Contour functions began at the upper margin of the aperture of the lips and proceeded clockwise around the vocal tract back to the point of origin. The *y* (anterior-posterior) and *z* (ventral-dorsal) coordinates were sampled at a set of equally spaced points along the vocal tract surface. In order to support statistical analysis of agreement measures across users, all contour functions were upsampled to match the number of data points in the largest vocal tract outline (153 data points in this dataset).

At each site, the disagreement among analysts was calculated as the mean Euclidean distance between the coordinates reported by each analyst and the group mean. This measure was calculated separately at each measurement site along the vocal tract outline, and was computed separately for experienced and inexperienced rtMRI users.

#### Outcome

**Fig. 8:**
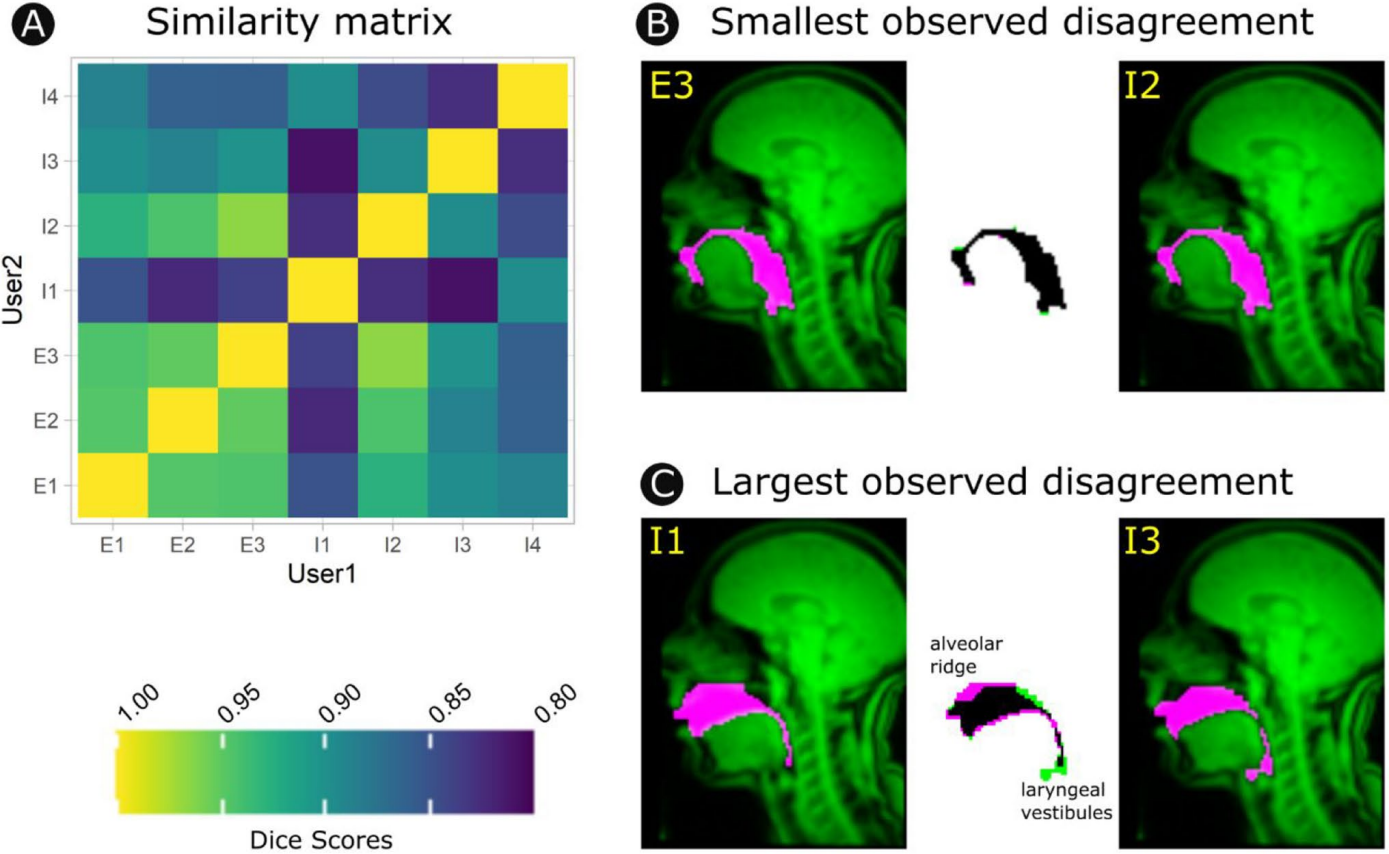
**A** Similarity matrix depicting Dice scores for each pair of users (E1–3 are experienced users, I1–4 are inexperienced). High values indicate greater similarity. The individual instances of the smallest disagreement (**B**) and the largest disagreement (**C**) as indicated by these comparisons. The central image of each montage depicts the area of agreement between vocal tract masks from the two users (black) as well as the areas of disagreement (pink/green). The flanking images depict the raw vocal tract for each user overlaid on the raw image for anatomical context. Even in images where large errors occurred, these were restricted to fixed structures, with broader agreement across the dynamic surface of the tongue, pharynx, and velum

Dice scores indicated agreement between users ranging from 0.81 to 0.97 (where 1 indicates complete agreement and 0 complete disagreement), with a tendency for agreement to be higher among users with greater experience with rtMRI data. While this range indicates considerable potential for between-user variation, a closer examination reveals that large disagreements did not reflect random variation, but rather diverging but legitimate analytical choices. In particular, disagreements were primarily driven by differences in how fixed structures were handled, while there was broad agreement on the boundaries of dynamic structures that are of primary interest to researchers, such as the tongue, velum, pharynx, and larynx (see Fig. 8).

The alveolar ridge is a structure posterior to the upper teeth that yields poor T1-weighted signal contrast with the boundaries of the vocal tract. Users adopted diverging strategies towards including/excluding pixels in this region, but were nonetheless internally consistent. Another case of diverging strategy was observed at the laryngeal vestibules. These comprise a space above the vocal folds of the larynx and below the epiglottis that may, or may not, be meaningful to include in the vocal tract depending on the research question. The relevance of this feature may also be conditional on the configuration of the tongue and the position of the larynx. For example, when the tongue is in a back position and the larynx raised, this space can become discontinuous with the remainder of the vocal tract. These issues highlight the importance of informing analyses with domain-specific knowledge of vocal anatomy, as well as the value of explicitly pre-specifying strategies to avoid cases of ambiguity.

**Fig. 9:**
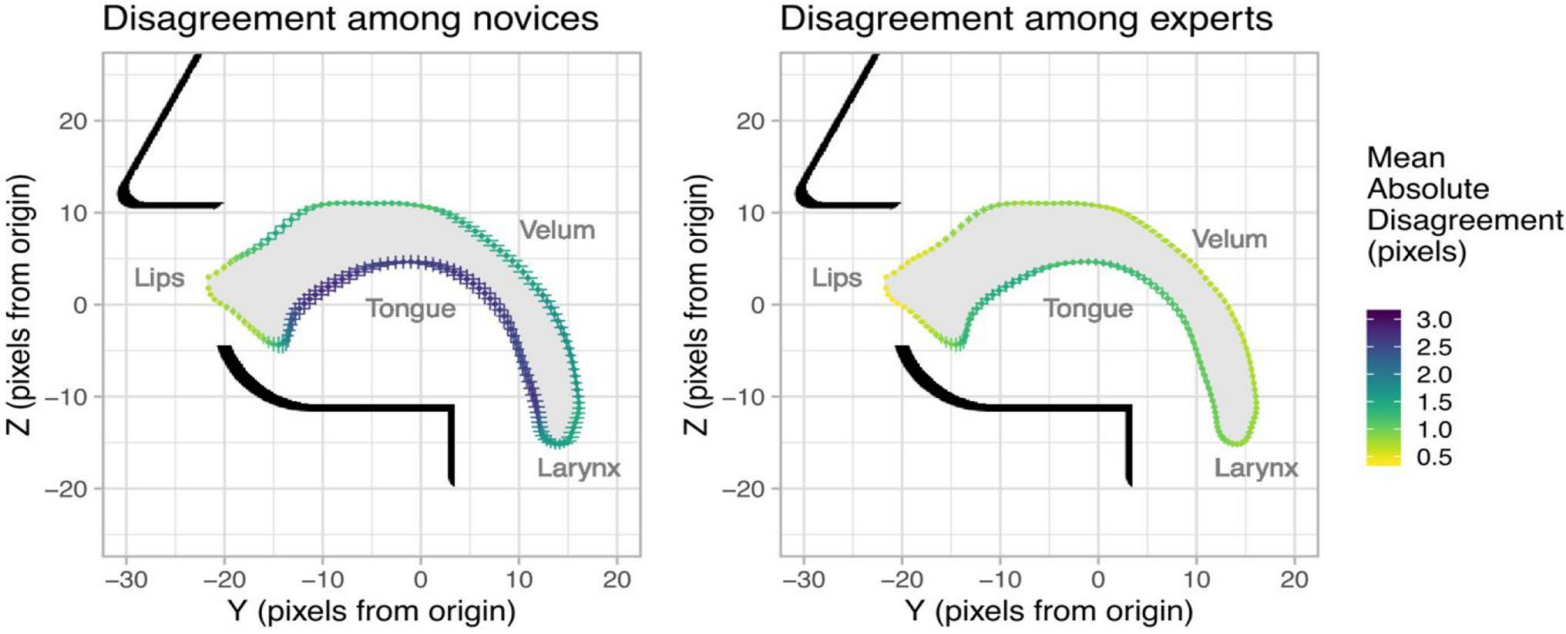
Disagreement in the coordinates of sites along the vocal tract outline among novices and among experts. Measurements are depicted at 153 equally spaced sites mapped onto the mean shape of the vocal tract. The mean absolute disagreement among analysts is depicted heuristically from cool (high disagreement) to warm (low disagreement) colours. The same information is depicted spatially as crosshatching so that readers may more intuitively judge the extent to which between-user disagreement meaningfully impacts these measurements.

A separate analysis of user disagreement across the outline of the vocal tract (see Fig. 9) demonstrated that disagreement among experienced users ranged from 0.4 to 1.5 pixels (1.0–3.5 mm), while disagreement among inexperienced users ranged from 0.7 to 2.7 pixels (1.75–6.75 mm). The larger of these figures is driven by differences in laryngeal vestibule strategies noted above. However, among experts, this disagreement rate constitutes, on average, a translation of one pixel, or the minimal observable disagreement for these data. It is noted that rates of small disagreements (i.e., those likely related to analysis variability rather than analytical strategy) occur most frequently at the surface of the tongue (see Fig. 9) where movements are largest.

## Discussion

We have tested a novel pipeline for processing dynamic images of the vocal tract as captured by rtMRI. Previous methodologies have implemented greater automation by leveraging deep learning techniques. However, these rely on training data sourced from a relatively narrow sample of behaviours, imaging centres, and pulse sequences (e.g., Asadiabadi & Erzin, 2020; Bresch & Narayanan, 2009; Eslami et al., 2020; Labrunie et al., 2018; van Leeuwen et al., 2019; Mannem & Ghosh, 2021; Pandey & Sabbir Arif, 2021; Ruthven et al., 2021; Silva & Teixeira, 2015; Somandepalli et al., 2017; Takemoto et al., 2019; Valliappan et al., 2019)**.** While our pipeline is more labour-intensive, we have demonstrated that it generalises beyond the dataset for which it was designed (see Belyk et al., 2022). Furthermore, we quantified the rates of disagreement between users, demonstrating that agreement was high among users with domain-relevant expertise.

In all cases but one (MB), analysts were using this pipeline for the first time, with minimal guidance beyond the written documentation, and with no opportunity to communicate or collaborate. Interestingly, the largest errors were observed at two points in the vocal tract where there is legitimate scope for disagreement in how appropriate it is to classify pixels as vocal tract. These were (1) the area between the tongue and the lower lip, where T1-weighted images have poor ability to distinguish the teeth and gingiva from air, and (2) a space just above and anterior to the larynx identified as the laryngeal vestibule, whose inclusion or exclusion should be dictated by the individual research question. A sound theoretically motivated and internally consistent strategy for handling these areas is expected to improve reliability.

### Limitations of the imaging modality

Real-time MRI constructs videos from T1-weighted images. The principal advantages of this approach are that (i) MRI can generate images in the midline of the vocal tract with little interference from surrounding tissue, (ii) T1-weighted images provide excellent tissue contrast between air and the soft tissue that surrounds the vocal tract, and (iii) the absence of ionising radiation makes the method safe for repeated use (Huettel et al., 2009). However, this imaging modality also has several limitations, some of which can be mitigated by supplementary pre-processing, and others which must moderate the interpretation of results.

### Intensity inhomogeneity

MRI data acquisition depends on producing a uniform magnetic field. However, placing objects within the bore of the scanner, even those with only weak magnetic properties such as human tissue, can introduce distortions. While many pulse sequences used for MRI data acquisition include corrective mechanisms such as magnetic shims, these corrections may remain imperfect (Gruetter & Boesch, 1992; Koch et al., 2009). The resulting inhomogeneity in the magnetic field can lead to inhomogeneity in the intensity of the resulting images. While local tissue contrasts remain sound, the same tissue type may produce a stronger signal at some points in space than others. Naturally, this poses an obstacle to tissue classification. Supplementary scripts are provided alongside this pipeline to implement inhomogeneity corrections, and analysts should consider carefully whether these may be necessary before further processing.

### Partial volume effects

This may be exacerbated by the hidden third dimension of the rtMRI image. The pixels in this modality are in fact three-dimensional voxels with a depth through the mid-sagittal plane usually on the order of several times the pixel width. This deeper sampling through the sagittal plane increases the volume over which the T1-signal is averaged, with corresponding benefits to the signal-to-noise ratio (SNR). However, there is also increased risk of inducing partial volume effects if the slice depth does not sample tissue uniformly. Pixels that span multiple tissue types or which contain air/tissue boundaries appear blurred (González Ballester et al., 2002). In some cases, these partial volume effects may present an obstacle to tissue classification as the distribution of intensity values in soft tissue, hard tissue, and air become less easily separable.

### Hard tissue is hard to image

T1-weighted images provide poor contrast between bone and air. This occurs because the T1-signal is proportional to the local abundance of hydrogen, which is abundant in soft tissue and sparse in both bone and air. Hence, while the teeth are certainly relevant structures to speech scientists, they are notably absent from these images. Likewise, the hard palate and the alveolar ridge are composed of a thin surface of soft tissue over a larger volume of bone and are not reliably identifiable in some datasets.

### Scalability

While this signal processing pipeline provides a considerable labour savings relative to a more manual approach, a careful analysis will still require some investment of time and expertise. However, the final stage of reviewing and validating individual images may become particularly tedious with very large samples. We note that such opportunities for manual correction are not common practice with existing methods (Kim et al., 2014), and researchers using our pipeline should choose to make pragmatic decisions about how extensively this is implemented.

While semi-automation through signal-processing undoubtedly increases the practical limits of dataset size, very large datasets may yet remain impractical. The authors have utilised this pipeline to analyse a moderately large dataset that included all frames in which 52 speakers produced audible vocal sound across multiple imaging runs (Belyk et al., 2022). In total, this dataset constituted more than 150,000 images and was found to be tractable for a single analyst. However, further developments that improve temporal resolution at data acquisition (e.g., Fu et al., 2015, 2017) may pose an increasing challenge to scalability.

### Future development

The development of fully automated rtMRI pipelines based on deep learning methods remains of considerable interest. However, practical deep learning solutions will require extensive training data far beyond that which is currently available. We suggest that the pipeline that we have presented could be used to generate human-validated training data on a sufficiently large scale to support the further advancement of deep learning models.

## Conclusions

We present a novel processing pipeline that provides a practical approach to taking quantitative measurements of dynamic movements of vocal tract underlying speech, singing, and expressions of emotion. We have demonstrated that the pipeline successfully generalises across datasets and produces consistent results across analysts, particularly if they have domain-relevant expertise. The latter is particularly important, as the method relies on providing knowledgeable analysts with iterative opportunities for manual intervention, correction, and validation of otherwise automated processes. This pipeline is openly available for immediate use by the scientific community. It is also envisioned that measurements produced by this pipeline could be used to provide a broader scope of training data to support the development of fully automated methods based on deep learning.

## References

[CR1] Asadiabadi S, Erzin E (2020). Vocal tract contour tracking in rtMRI using deep temporal regression network. IEEE/ACM Transactions on Audio Speech and Language Processing.

[CR2] Belyk M, McGettigan C (2022). Real-time magnetic resonance imaging reveals distinct vocal tract configurations during spontaneous and volitional laughter. Philosophical Transactions of the Royal Society B: Biological Sciences.

[CR3] Belyk M, Schultz BG, Correia J, Beal DS, Kotz SA (2019). Whistling shares a common tongue with speech: Bioacoustics from real-time MRI of the human vocal tract. Proceedings of the Royal Society B.

[CR4] Belyk M, Waters S, Kanber E, Miquel ME, McGettigan C (2022). Individual differences in vocal size exaggeration. Scientific Reports.

[CR5] Boersma, P., & Weenink, D. (2019). *Praat: Doing phonetics by computer*. http://www.praat.org/. Accessed 05/11/2021

[CR6] Bresch E, Narayanan S (2009). Region segmentation in the frequency domain applied to upper airway real-time magnetic resonance images. IEEE Transactions on Medical Imaging.

[CR7] Carey D, Miquel ME, Evans BG, Adank P, McGettigan C (2017). Functional brain outcomes of L2 speech learning emerge during sensorimotor transformation. NeuroImage.

[CR8] Carignan C, Hoole P, Kunay E, Pouplier M, Joseph A, Voit D, Frahm J, Harrington J (2020). Analyzing speech in both time and space: Generalized additive mixed models can uncover systematic patterns of variation in vocal tract shape in real-time MRI. Laboratory Phonology.

[CR9] Dice LR (1945). Measures of the amount of ecologic association between species. Ecology.

[CR10] Echternach M, Sundberg J, Markl M, Richter B (2010). Professional opera tenors’ vocal tract configurations in registers. Folia Phoniatrica et Logopaedica.

[CR11] Echternach M, Sundberg J, Baumann T, Markl M, Richter B (2011). Vocal tract area functions and formant frequencies in opera tenors’ modal and falsetto registers. The Journal of the Acoustical Society of America.

[CR12] Echternach M, Popeil L, Traser L, Wienhausen S, Richter B (2014). Vocal tract shapes in different singing functions used in musical theater singing-a pilot study. Journal of Voice.

[CR13] Eslami M, Neuschaefer-Rube C, Serrurier A (2020). Automatic vocal tract landmark localization from midsagittal MRI data. Scientific Reports.

[CR14] Fant, G. (1960). *Acoustic theory of speech production*. Mouton.

[CR15] Fu M, Zhao B, Carignan C, Shosted RK, Perry JL, Kuehn DP, Liang Z-P, Sutton BP (2015). High-resolution dynamic speech imaging with joint low-rank and sparsity constraints. Magnetic Resonance in Medicine.

[CR16] Fu M, Barlaz MS, Holtrop JL, Perry JL, Kuehn DP, Shosted RK, Liang Z-P, Sutton BP (2017). High-frame-rate full-vocal-tract 3D dynamic speech imaging. Magnetic Resonance in Medicine.

[CR17] González Ballester MÁ, Zisserman AP, Brady M (2002). Estimation of the partial volume effect in MRI. Medical Image Analysis.

[CR18] Goodfellow, I., Bengio, Y., & Courville, A. (2016). *Deep Learning*. Massachusetts Institute of Technology. https://www.deeplearningbook.org/

[CR19] Gruetter R, Boesch C (1992). Fast, noniterative shimming of spatially localized signals. In vivo analysis of the magnetic field along axes. Journal of Magnetic Resonance (1969).

[CR20] Haast RAM, Ivanov D, Uludağ K (2018). The impact of correction on MP2RAGE cortical T1 and apparent cortical thickness at 7T. Human Brain Mapping.

[CR21] Hart PE, Nilsson NJ, Raphael B (1968). A formal basis for the heuristic determination of minimum cost paths. IEEE Transactions on Systems Science and Cybernetics.

[CR22] Huettel, S., Song, A., & McCarthy, G. (2009). *Functional Magnetic Resonance Imaging* (2nd ed.). Sinauer Associates Inc.

[CR23] Isaieva K, Laprie Y, Leclère J, Douros IK, Felblinger J, Vuissoz P-A (2021). Multimodal dataset of real-time 2D and static 3D MRI of healthy French speakers. Scientific Data.

[CR24] Kim, J., Kumar, N., Lee, S., & Narayanan, S. (2014). Enhanced airway-tissue boundary segmentation for real-time magnetic resonance imaging data. *Proceedings of the 10th International Seminar on Speech Production, ISSP 2014*, *i*, 1–4.

[CR25] Koch KM, Rothman DL, de Graaf RA (2009). Optimization of static magnetic field homogeneity in the human and animal brain in vivo. Progress in Nuclear Magnetic Resonance Spectroscopy.

[CR26] Labrunie M, Badin P, Voit D, Joseph AA, Frahm J, Lamalle L, Vilain C, Boë LJ (2018). Automatic segmentation of speech articulators from real-time midsagittal MRI based on supervised learning. Speech Communication.

[CR27] Lammert, A., Proctor, M., Katsamanis, A., & Narayanan, S. (2011). Morphological variation in the adult vocal tract: A modeling study of its potential acoustic impact. *Proceedings of the Annual Conference of the International Speech Communication Association, INTERSPEECH*, *August*, 2813–2816. 10.21437/interspeech.2011-704

[CR28] Lim Y, Toutios A, Bliesener Y, Tian Y, Lingala SG, Vaz C, Sorensen T, Oh M, Harper S, Chen W, Lee Y, Töger J, Monteserin ML, Smith C, Godinez B, Goldstein L, Byrd D, Nayak KS, Narayanan SS (2021). A multispeaker dataset of raw and reconstructed speech production real-time MRI video and 3D volumetric images. Scientific Data.

[CR29] Lynn E, Narayanan SS, Lammert AC (2021). Dark tone quality and vocal tract shaping in soprano song production: Insights from real-time MRI. JASA Express Letters.

[CR30] Mannem R, Ghosh PK (2021). A deep neural network based correction scheme for improved air-tissue boundary prediction in real-time magnetic resonance imaging video. Computer Speech & Language.

[CR31] Miller NA, Gregory JS, Aspden RM, Stollery PJ, Gilbert FJ (2014). Using active shape modeling based on MRI to study morphologic and pitch-related functional changes affecting vocal structures and the airway. Journal of Voice.

[CR32] Mills N, Lydon A-M, Davies-Payne D, Keesing M, Geddes DT, Mirjalili SA (2020). Imaging the breastfeeding swallow: Pilot study utilizing real-time MRI. Laryngoscope Investigative Otolaryngology.

[CR33] Narayanan S, Toutios A, Ramanarayanan V, Lammert A, Kim J, Lee S, Nayak K, Kim Y-C, Zhu Y, Goldstein L, Byrd D, Bresch E, Ghosh P, Katsamanis A, Proctor M (2014). Real-time magnetic resonance imaging and electromagnetic articulography database for speech production research. The Journal of the Acoustical Society of America.

[CR34] Olthoff A, Zhang S, Schweizer R, Frahm J (2014). On the physiology of normal swallowing as revealed by magnetic resonance imaging in real time. Gastroenterology Research and Practice.

[CR35] Pandey, L., & Sabbir Arif, A. (2021). Silent speech and emotion recognition from vocal tract shape dynamics in real-time MRI. *ACM SIGGRAPH 2021 Posters*, 1–2. 10.1145/3450618.3469176

[CR36] Proctor M, Bresch E, Byrd D, Nayak K, Narayanan S (2013). Paralinguistic mechanisms of production in human “beatboxing”: A real-time magnetic resonance imaging study. The Journal of the Acoustical Society of America.

[CR37] Ramsay, J. O., Hooker, G., & Spencer, S. (2009). *Functional Data Analysis With R and MATLAB*. Springer Science+Busness Media Inc.

[CR38] Ramsay, J. O., Wickham, H., Graves, S., & Hooker, G. (2017). *fda: Functional Data Analysis*. https://cran.r-project.org/package=fda. Accessed 11/16/2017

[CR39] Ruthven M, Miquel ME, King AP (2021). Deep-learning-based segmentation of the vocal tract and articulators in real-time magnetic resonance images of speech. Computer Methods and Programs in Biomedicine.

[CR40] Silva S, Teixeira A (2015). Unsupervised segmentation of the vocal tract from real-time MRI sequences. Computer Speech and Language.

[CR41] Sled, J. G., & Pike, G. B. (1998). Understanding intensity non-uniformity in MRI. In W. Wells, A. Colchester, & S. Delp (Eds.), *Medical Image Computing and Computer-Assisted Intervention—MICCAI’98. MICCAI 1998. Lecture Notes in Computer Science,* (Issue 1496, pp. 614–622).

[CR42] Somandepalli, K., Toutios, A., & Narayanan, S. S. (2017). Semantic edge detection for tracking vocal tract air-tissue boundaries in real-time magnetic resonance images. *Interspeech*, 631–635. 10.21437/Interspeech.2017-1580

[CR43] Takemoto, H., Goto, T., Hagihara, Y., Hamanaka, S., Kitamura, T., Nota, Y., & Maekawa, K. (2019). Speech organ contour extraction using real-time MRI and machine learning method. *Proceedings of the Annual Conference of the International Speech Communication Association, INTERSPEECH*, *2019-Septe*, 904–908. 10.21437/Interspeech.2019-1593

[CR44] Titze IR (2008). Nonlinear source–filter coupling in phonation: Theory. The Journal of the Acoustical Society of America.

[CR45] Valliappan CA, Kumar A, Mannem R, Karthik GR, Ghosh PK (2019). An improved air tissue boundary segmentation technique for real time magnetic resonance imaging video using segnet. *ICASSP, IEEE International Conference on Acoustics, Speech and Signal Processing - Proceedings*, *2019-May*.

[CR46] van Leeuwen KG, Bos P, Trebeschi S, van Alphen MJA, Voskuilen L, Smeele LE, van der Heijden F, van Son RJJH (2019). CNN-based phoneme classifier from vocal tract MRI learns embedding consistent with articulatory topology. Interspeech.

[CR47] Waters S, Kanber E, Lavan N, Belyk M, Carey D, Cartei V, Lally C, Miquel M, Mcgettigan C, Mcgettigan C (2021). Singers show enhanced performance and neural representation of vocal imitation. Philosophical Transactions of the Royal Society B: Biological Sciences.

[CR48] Wiltshire CEE, Chiew M, Chesters J, Healy MP, Watkins KE (2021). Speech movement variability in people who stutter: A vocal Tract magnetic resonance imaging study. Journal of Speech, Language, and Hearing Research.

[CR49] Zhang S, Block KT, Frahm J (2010). Magnetic resonance imaging in real time: Advances using radial FLASH. Journal of Magnetic Resonance Imaging.

[CR50] Zhang S, Olthoff A, Frahm J (2012). Real-time magnetic resonance imaging of normal swallowing. Journal of Magnetic Resonance Imaging.

